# Preparation and Characterization of Carbazole-Based Luminogen with Efficient Emission in Solid and Solution States

**DOI:** 10.3390/ma16114193

**Published:** 2023-06-05

**Authors:** Beibei Zhang, Lingzhong Wei, Xuansi Tang, Zizhan Jiang, Song Guo, Linmin Zou, Huihong Xie, Yongyang Gong, Yuanli Liu

**Affiliations:** Guangxi Key Laboratory of Optical and Electronic Materials and Devices, College of Materials Science and Engineering, Guilin University of Technology, Guilin 541004, China

**Keywords:** organic luminogens, carbazole, dual state emission, high efficiency

## Abstract

Organic luminescent materials with high luminescence efficiency in both solution and solid states, namely dual-state emission (DSE), have attracted considerable attention due to their promising applications in various fields. In order to enrich the variety of DSE materials, carbazole, similar to triphenylamine (TPA), was utilized to construct a novel DSE luminogen named 2-(4-(9H-carbazol-9-yl)phenyl)benzo[d]thiazole (CZ-BT). CZ-BT exhibited DSE characteristics with fluorescence quantum yields of 70, 38 and 75% in solution, amorphous and crystalline states, respectively. CZ-BT shows thermochromic and mechanochromic properties in solution and solids, respectively. Theoretical calculations show that there is a small conformational difference between the ground state and the lowest singly excited state of CZ-BT and that it exhibits a low non-radiative transition characteristic. The oscillator strength during the transition from the single excited state to the ground state reaches 1.0442. CZ-BT adopts a distorted molecular conformation with intramolecular hindrance effects. The excellent DSE properties of CZ-BT can be explained well using theoretical calculations and experimental results. In terms of application, the CZ-BT has a detection limit for the hazardous substance picric acid of 2.81 × 10^−7^ mol/L.

## 1. Introduction

Organic luminophores exhibiting intense emission in both solid and solution states have attracted considerable attention due to their wide applications in organic light-emitting diodes (OLEDs), chemical sensors, bioimaging, etc. [1,2,3,4,5,6,7,8,9]. Conventional organic luminophores containing planar aromatic rings exhibit efficient luminescence in dilute solutions, but there is weak or no emission in concentrated solutions or aggregated states, which is known as the aggregation-caused quenching (ACQ) phenomenon. In 2001, Tang’s group discovered that compounds with propeller structures, such as 1-methyl-1,2,3,4,5-pentaphenylsilane and tetraphenylethylene (TPE), exhibited the optical phenomenon of anti-ACQ and termed it aggregation-induced emission (AIE) [10]. AIE-active luminophores exhibit bright emission in the aggregated state and weak or no emission in the solution state. The AIE mechanism was mainly attributed to the restriction of intramolecular rotation in the aggregated state. In the past two decades, AIE-active compounds have been widely synthesized and applied in various areas, such as chemical/biosensors and bioimaging, OLEDs, solar cells and optical devices as well as others [11,12,13,14,15,16].

Recently, numerous endeavors have been made to circumvent the limitations of ACQ- and AIE-type luminescent materials, and to achieve efficient emission both in solution and in the solid state, which has been termed the dual-state emission (DSE) characteristic. Yuan et al. reported a series of triphenylene-substituted triphenylene analogues displaying DSE properties with high quantum yields in THF solution (73%) and crystals (87.1%) via the strategy of conjugation-induced rigidity [8]. Wang et al. reported diarylmaleimides (DAMs) with 2- or 3-substituted-benzofuran moiety, which showed good DSE behaviors [17]; however, the emission of sulfur derivatives was quenched either in solution or in the solid state. Eisler et al. reported a series of 3,4-thioaryl-N-propylmaleimide derivatives with quantum yields ranging from 2% to 80% in solution and from 2% to 87% in the solid state [18]. Dong et al. reported that attaching benzene rings at different positions in pyrrole can lead to a balance between distorted conformation and effective conjugation in the compound. Notably, a decrease in emission in solution was observed when the phenyl ring was attached to the nitrogen atom. Phenyl-substituted pyrrole luminescence at positions 2 and 5 has the highest luminescence efficiency in solution and solid state [19].

To date, organic luminophores with DSE properties have rarely been studied in depth in terms of type, amount and mechanism compared to AIE- or ACQ-active luminogens [1]. Previously, we combined the Einstein radiation transition formula with photoluminescence efficiency to illustrate the mechanism of DSE luminophores based on triphenylamine (TPA) at the quantum chemical level. Carbazole is similar to triphenylamine in chemical structure and is widely used as a basic unit for the construction of organic luminescent materials [20,21,22], but the obtained luminescent materials have either AIE activity [23,24] or effective luminescence in dilute solutions. Carbazole derivatives reported in recent literature, which can effectively emit in both solution and solid state, are still rare [25,26,27], especially those obtained from combining theoretical calculations and experimentally relevant literatures. Therefore, it is worthwhile to thoroughly investigate whether planarized carbazole can avoid strong π–π interactions and achieve DSE properties in both solution and solid state.

**Figure 1:**
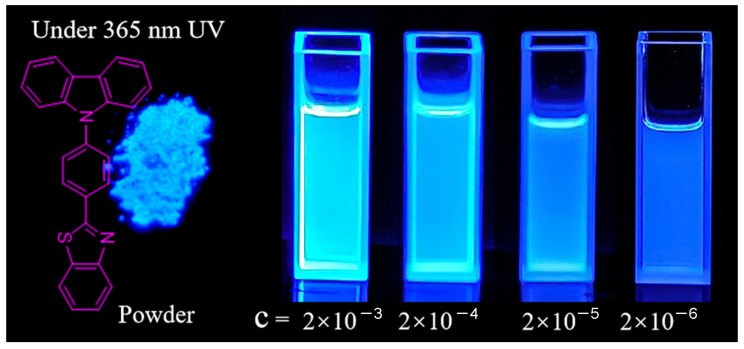
Chemical structure of CZ-BT and its luminescent photographs in solids and in DCM solutions of different concentrations under excitation with 312 nm UV light; the unit of solution concentration = mol/L.

Herein, we designed and synthesized a twisted D-A compound containing carbazole and a benzothiazole group, named 2-(4-(9H-carbazol-9-yl)phenyl)benzo[d]thiazole (CZ-BT), via the Suzuki reaction, whose quantum yields are up to 75 and 70% in crystalline and solution states, respectively (Figure 1). CZ-BT can achieve high-efficiency luminescence in the solid state due to the twisted configuration of planar molecules, which can effectively avoid the strong π–π interaction in its aggregate state. Theoretical calculations showed that the root mean square deviation (RMSD) between the ground and excited configurations of CZ-BT is very small, the S_1_ transition excitation oscillator intensity is high and the intramolecular site hindrance effect is significant, allowing CZ-BT to emit efficient luminescence in dilute solutions. In addition, the CZ-BT compound can be used for the detection of picric acid.

## 2. Results and Discussion

### 2.1. Synthesis

CZ-BT was obtained via the well-known Suzuki C–C coupling reaction between 2-bromobenzothiazole and 4-(9H-carbazol-9-yl)phenyl)boronic acid with palladium catalyst in a high yield of 80%. CZ-BT has good solubility in common organic solvents. The chemical structure of CZ-BT was characterized using NMR spectra (Figures S1 and S2), high-resolution mass spectra (HRMS) (Figure S3) and single-crystal analysis (Figure S4, Table S1 and CCDC 1952420).

### 2.2. Photophysical Properties in Solution

**Figure 2:**
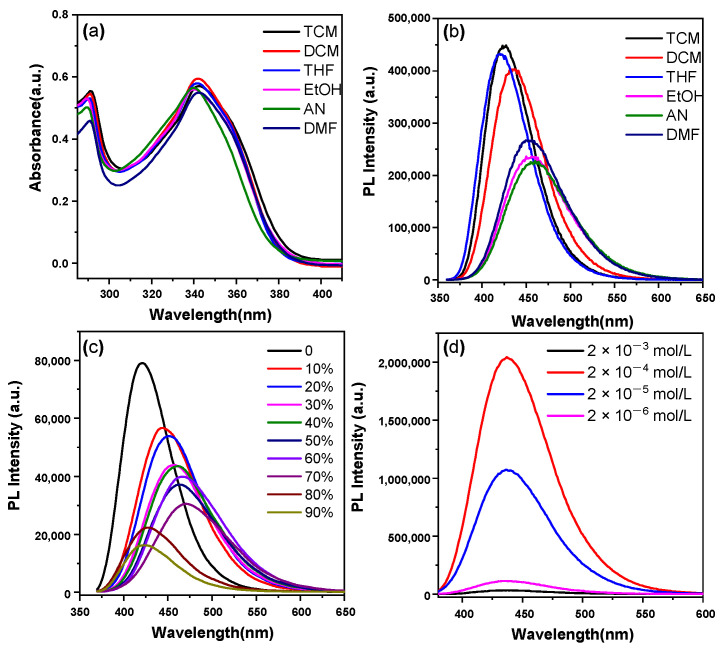
Absorption (**a**) and emission (**b**) spectra of CZ-BT in different organic solvents; emission spectra of CZ-BT in THF and THF–water mixtures with varying water fractions (*f*_w_) (**c**); fluorescence intensity of different concentrations of CZ-BT in DCM solution (**d**). Concentration: 20 μM (**a**–**c**); excitation wavelength: 320 nm.

CZ-BT exhibits two absorption bands at around 290 and 340 nm in different solvents; the former and the latter can be assigned to the π–π* transition and the intramolecular charge transfer (ICT) transition, respectively (Figure 2a). The photoluminescence (PL) showed a slight red-shift and the luminescence intensity decreased with the increasing of solvent polarity, peaking at 457, 424, 420, 434, 460 and 454 nm in ethanol (EtOH), trichloromethane (TCM), tetrahydrofuran (THF), dichloromethane (DCM), acetonitrile (AN) and dimethylformamide (DMF), respectively. The absolute photoluminescence quantum efficiency (PLQY) value of CZ-BT in DCM is up to 70%. The relative PLQY values of CZ-BT in EtOH, TCM, THF, AN and DMF are 43, 37, 49, 30 and 37.9%, respectively. These results are highly indicative of the ICT feature of CZ-BT and efficient emission in solution states.

Organic luminophores with ACQ properties lead to luminescence quenching with increasing concentration. To verify the DSE properties, the luminescence behavior of CZ-BT in solution at different concentrations was carefully studied. As shown in Figure 2d, the bright emission of CZ-BT solutions at a concentration of 2.0 × 10^−3^ mol/L suggests that CZ-BT does not have ACQ properties at high concentrations. On the other hand, the effective emission in solution at low concentration (2.0 × 10^−6^ mol/L) suggests that CZ-BT can overcome the disadvantages of AIE, which usually shows weak or no emission in solution. The emission peak showed no obvious red-shift with a significant increase in concentration, indicating that CZ-BT did not form strong π–π interactions even at high concentrations.

It is worth noting that CZ-BT showed the highest brightness when at the highest concentration, according to the fluorescence photographs. In fact, when a highly concentrated solution is irradiated with excitation light in the cuvette, the effective absorption for the sample may be limited only to a certain thickness. In addition, the emitted photons will be absorbed by other dissolved molecules in the solution before reaching the detector, resulting in a reduction in the intensity of the fluorescence spectrum of the highly concentrated sample, known as the self-absorption phenomenon. It is well known in the field of luminescent materials research [28].

### 2.3. The Effect of Temperature on the Optical Properties in Solutions

**Figure 3:**
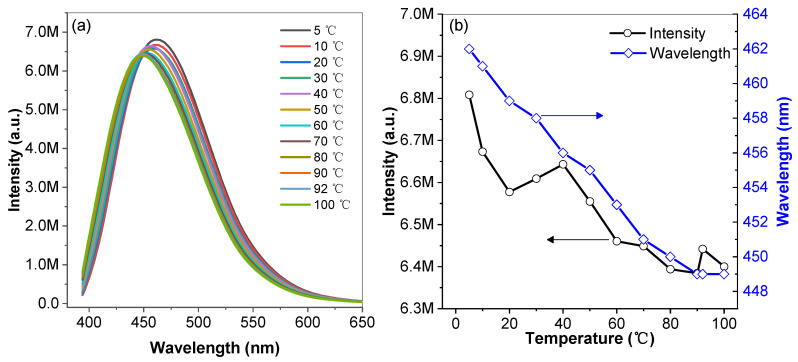
Emission spectra of DMF solutions of CZ-BT at different temperatures (**a**); fluorescence intensity and emission wavelength versus temperature (**b**) of CZ-BT in DMF solution. Concentration = 20 μM, excitation wavelength = 320 nm.

Temperature can significantly affect the photophysical properties of organic luminogens [29]. We performed a temperature-dependent experiment using a programmed temperature method. Photoluminescence (PL) spectra were collected sequentially with the increasing of temperature after the system was equilibrated for 10 min at each temperature setting value. The fluorescence intensity decreased with the increase in temperature, due to the increase in vibrational deactivation. At the same time, the maximum emission peak of the CZ-BT solution gradually exhibited a remarkable blue-shift from 462 nm (5 °C) to 449 nm (100 °C), as shown in Figure 3a, corresponding to the change of Commission Internationale de L’Eclairage (CIE) coordinates from (0.1606, 0.1936) to (0.1563, 0.1488) in Figure S5 and Table S2. It is relatively rare for organic luminescent materials to exhibit a blue-shift in luminescence with increasing temperature. The mechanism maybe due to the increase of dihedral angle between the intermediate benzene ring and the carbazole unit, resulting in a slightly reduced degree of conjugation and a blue-shift of the luminescence of the molecular system [30].

### 2.4. Optical Properties CZ-BT at Solid States

**Figure 4:**
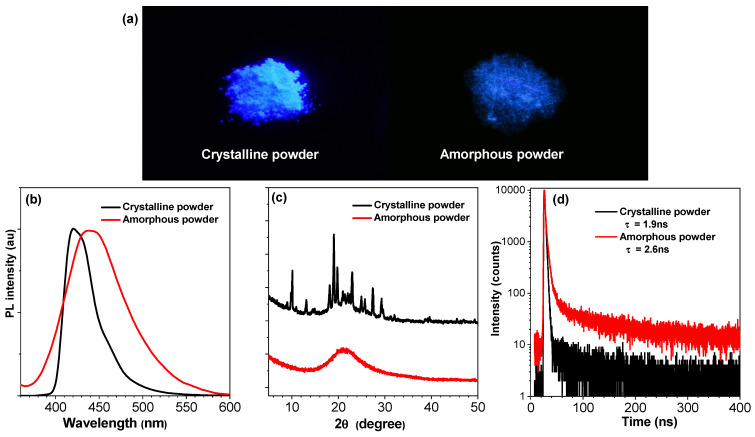
Emission photos (**a**), emission spectra (**b**), XRD curves (**c**) and lifetime decay curves (**d**) of CZBT in crystalline and amorphous states.

Unlike macromolecules, solid samples of organic small molecules can be divided into crystalline samples and amorphous samples. Usually, organic luminescent materials exhibit significantly different optical properties due to the differences in molecular interactions in crystalline and amorphous samples. As shown in Figure 4, the crystalline powder of CZ-BT exhibits sharp and intense diffraction peaks and its emission peaks are located at 419 nm with a PLQY value of up to 75%. However, the amorphous sample of CZ-BT shows broad diffuse halos with low intensity, reflecting their disordered amorphous nature. The emission peak and PLQY of the amorphous sample of CZ-BT were 439 nm and 38%, respectively. Thus, CZ-BT shows high luminescence efficiency in both crystalline and amorphous phases. Correspondingly, time-resolved spectra show that lifetimes of crystalline and amorphous samples of CZ-BT are 1.9 ns and 2.6 ns, respectively. Notably, organic crystalline samples can be transformed into an amorphous state under force, and reversible recovery can also be achieved under heating or solvent fumigation, suggesting that CT-BT can be used as a smart material for force-stimulated fluorescence response. Furthermore, the full width at half maximum of the fluorescence emission spectrum of CZ-BT crystalline solids is only 38 nm, which demonstrates potential applications in solid-state lasers [31].

### 2.5. Single Crystals and Theory Calculations

**Figure 5:**
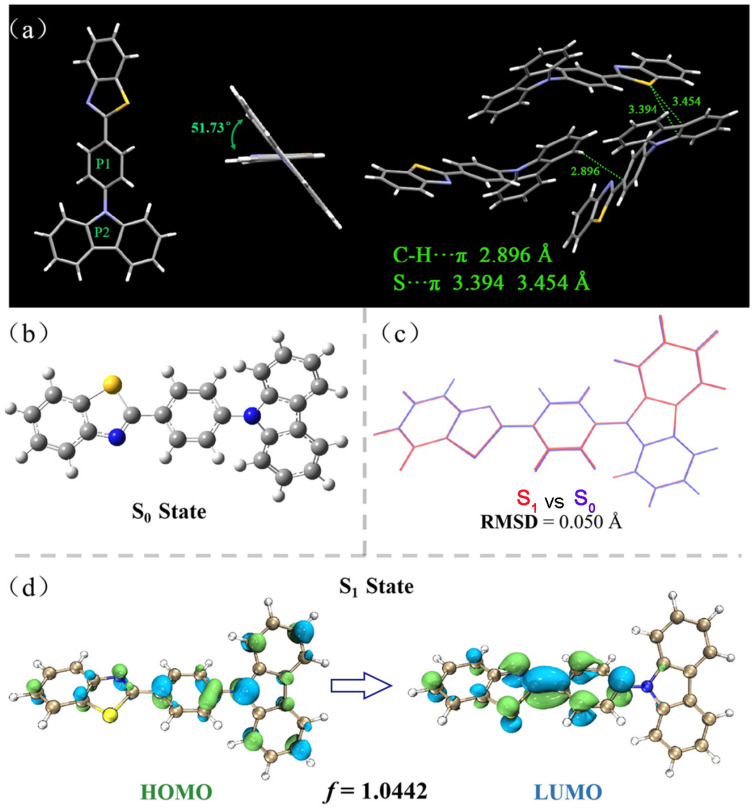
The single-crystal structure of CZ-BT and the intermolecular interactions in the single crystal (**a**); the conformation of the ground state (**b**) and the conformational differences (**c**) between the ground state and the lowest excited state of CZ-BT obtained via theoretical calculations; molecular orbital amplitude plots of HOMO and LUMO energy levels of CZ-BT (**d**).

Then, single-crystal and theoretical calculations were carried out to further understand the DSE characteristics of CZ-BT. Single crystals of CZ-BT were carefully presented (Figure 5 and Figure S4, Table S1 and CCDC number: 1952420), and abundant and effective intermolecular interactions could be clearly observed. In single crystals of CZ-BT, C-H···π (2.896 Å) and S···π (3.394, 3.454 Å) short contacts were found. A large number of intermolecular interactions in the crystals greatly impede the molecular motions to provide rigid conformations. The dihedral angle of P1 and P2 is 51.73°; this result indicates the twisted molecular configuration of CZ-BT, which can effectively inhibit the quenching of luminescence in solution or solid states caused by the formation of strong π–π interactions.

To understand the underlying DSE mechanism, density functional theory (DFT) and time-dependent density functional theory (TD-DFT) were used to investigate the S_0_ and S_1_ geometries based on the M06-2X/def-TZVP level using the Gaussian 16 B.01 software package [32]. In the S_0_ state, CZ-BT adopted twisted conformation with a 50.50° degree dihedral angle between the central benzene ring and the carbazole in the gas phase, which is consistent with the corresponding dihedral angle in the single crystal structure.

A real space function called the Interaction Region Indicator (IRI) [33] was used to assess the rigidity of the molecule. As shown in Figure S6, the IRI shows not only the chemical bonds but also the intramolecular interactions. Steric hindrance effects and van der Waals effects between the central benzene ring, carbazole and benzothiazole hinder the intramolecular rotation of CZ-BT and reduce non-radiative transitions, thus increasing the luminescence efficiency in solution. These results are consistent with the high luminescence in solution and suggest that CZ-BT is a typically rigid molecule.

In general, the conformational difference between the ground and excited states of rigidly structured fluorophores can be measured using the root mean square deviation (RMSD). The conformational difference between the ground state (S_0_) and excited state (S_1_) of CZ-BT is only 0.050 Å; the smaller the RMSD, the smaller the non-radiative transition [34].

The molecular orbitals (MOs) and calculated energy levels are shown in Figure 5d. The highest occupied molecular orbital (HOMO) of the CZ-BT is mainly located on the electron-donating CZ units; however, the lowest unoccupied molecular orbital (LUMO) is dominated by orbitals from the electron-accepting BT. Generally, such electron distribution imparts the dye molecules with an intrinsic intramolecular charge transfer property, in accordance with the experimental data. Furthermore, the oscillator strength is up to 1.0442, which is a dimensionless quantity which expresses the probability of absorption or emission of electromagnetic radiation in transitions between different energy levels of the molecule. The equation for the quantum yield of fluorescence based on Einstein’s radiation transitions shows that oscillator strength is proportional to the efficiency of the luminescence [34]. In addition, typical AIE-active compounds generally have a small oscillator strength, in contrast to compounds that emit efficiently in dilute solutions. The twisted molecular conformation and the high oscillator intensity allow CZ-BT to exhibit a unique DSE behavior. The theoretical calculations are in good agreement with the experimental phenomena.

### 2.6. Explosive Detection

**Figure 6:**
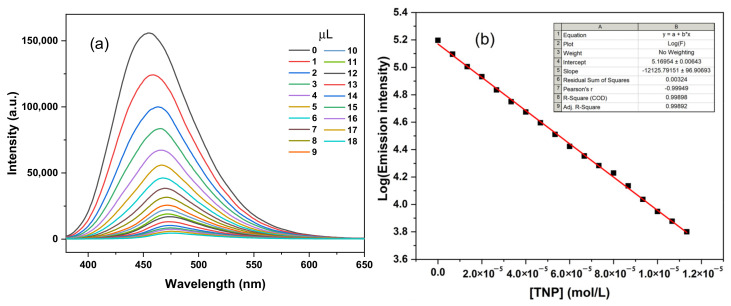
(**a**) PL spectra of CZ-BT DMF solutions (2 uM/mL) with different concentrations of TNP (λex = 350 nm); (**b**) emission intensity of CZ-BT solution against PA content.

Picric acid (PA) is an organic compound mainly used in the manufacture of explosives and dyes. PA is known to be toxic to humans, and inhalation or exposure to excessive amounts can cause headaches, nausea and breathing difficulties [35,36,37]. PA is also harmful to the environment and can affect ecological balance and water safety if it enters water bodies or soil. Fluorescence sensors are an attractive method for PA detection due to their low cost, fast response and good portability [38]. Compounds with DSE activity are ideal explosive detection sensors for the detection of PA. To verify the ability of CZ-BT with DSE activity to detect PA, we investigated the identification of PA in solution. As shown in Figure 6, fluorescence titration was performed to monitor the detection capability. In fluorescence titration with the gradual addition of PA (0–18 μM), the fluorescence intensity of CZ-BT (2 μM) is quenched slowly with the increase of PA and exhibits a good linear curve between fluorescence intensity and PA concentration.

## 3. Conclusions

In summary, a twisted dual-state emission luminogen named CZ-BT combined with carbazole and benzothiazole was designed and synthesized successfully through a Suzuki reaction, and its chemical structure and photophysical properties were investigated. CZ-BT has an absolute PLQY of 70, 75 and 38% in solution, crystalline and amorphous forms, respectively. The fluorescence emission peak of this compound red-shifts with increasing solvent polarity and gradually blue-shifts with increasing temperature, suggesting the solvent and temperature dependence of the fluorescence emission. Theoretical calculations reveal small differences in the configuration of the S_0_ and S_1_ of CZ-TB, and large oscillator strengths for the S_1_ → S_0_ transitions; in addition, there is a significant potential hindrance effect and twisted conformation within the CZ-BT molecule. At last, this work will provide a reference for designing novel DSE compounds, and we expect more and more DSE materials with high quantum efficiency to be reported in the future.

## Data Availability

Data is contained within the article.

## Supplementary Materials

The following supporting information can be downloaded at: https://www.mdpi.com/article/10.3390/ma16114193/s1, Figure S1: ^1^H NMR spectrum of CZ-BT; Figure S2: ^13^C NMR spectrum of CZ-BT; Figure S3: HRMS spectrum of CZ-BT; Figure S4: An ORTEP drawing of CZ-BT. Thermal ellipsoids are shown at the 50% probability level; Figure S5: CIE coordinates of DMF solution of CZ-BT at 5 and 100 °C; Figure S6: Intramolecular potential hindering effect of CZ-BT; Table S1: Crystal data and structure refinement for CZ-BT; Table S2: Colour coordinates of CZ-BT solutions at different temperatures.
