# Supplementary material for: Preparation and Characterization of Carbazole-Based Luminogen with Efficient Emission in Solid and Solution States
Source: Materials (Basel). 2023 Jun 5;16(11):4193. doi: 10.3390/ma16114193 (PMC10254887; doi:10.3390/ma16114193)
Supplement: Supplementary file 1 [file materials-16-04193-s001.zip › materials-2396367-supplementary/Supplementary.pdf]

materials

Support information

Preparation and characterization of carbazole-based luminogen with efficient emission in solid and solution states

Beibei Zhang, Lingzhong Wei, Xuansi Tang, Zizhan Jiang, Song Guo, Linmin Zou*, Huihong Xie, Yongyang Gong* and Yuanli Liu

Guangxi Key Laboratory of Optical and Electronic Materials and Devices, College of Materials Science and Engineering, Guilin University of Technology, No.12 Jian'gan Rd., Qixing District, Guilin 541004, China.
* Correspondence: gordon_best@qq.com (L.Z); yygong@glut.edu.cn (Y.G.)

Abstract: Organic luminescent materials with high luminescent efficiency in both solution and solid states, namely dual-state emission (DSE), have attracted considerable attentions due to their promising applications in various fields. In order to enrich the variety of DSE materials, carbazole, similar to triphenylamine (TPA), was utilized to construct a novel DSE luminogen named 2-(4-(9H-carbazol-9-yl)phenyl)benzo[d]thiazole (CZ-BT). CZ-BT exhibited DSE characteristics with fluorescence quantum yields of 75, 38 and 66.8% in solution, amorphous and crystalline states, respectively. CZ-BT shows thermochromic and mechanchromic properties in solution and solids, respectively. Theoretical calculations show that there is a small conformational difference between the ground state and the lowest singly excited state of CZ-BT and exhibiting a low non-radiative transition characteristic. The oscillator strength during the transition from the singly excited state to the ground state reaches 1.0442. CZ-BT adopts a distorted molecular conformation with intramolecular hindrance effects. The excellent DSE properties of CZ-BT can be well explained by theoretical calculations and experimental results. In terms of application, the CZ-BT has a detection limit for hazardous substances picric acid of 2.81×10^{-7} mol/L.

Keywords: organic luminogens; carbazole; dual state emission; high-efficiency

Experimental details

1 Instruments

^{1}H NMR (500 MHz) and ^{13}C NMR (126 MHz) spectra were recorded on a Bruker AMX-500 NMR spectrometer in deuterated solvent at room temperature, and chemical shifts were reported in ppm relative to tetramethylsilane (TMS, δ = 0 ppm). Mass spectrum was recorded on a Bruker MALDI Biotyper or Bruker Q-Exactive Thermofisher. Absorption measurements were performed on a UV3600 UV-Vis spectrophotometer. The emission spectra, solids quantum yields and lifetime were recorded on a FluoroMax-4 fluorescence spectrophotometer. Quantum yields (Φ) of solution were estimated using quinine sulphate (Φ = 54% in 0.1 N H_2SO_4) as standard, while solid-state efficiencies were determined by an integrating sphere.

The fluorescence lifetime measurement took-place on a Delta Flex-Moduler Fluorescence Life-time system (Horiba Scientific, UK) with the time-correlated single-photon counting (TCSPC) procedure. The fluorescence lifetime calculation was done by the following equation:

$$F(t) = \sum_{i=1}^{n} ai\ \exp(-t/\tau i)$$

where the number of segregate decay components was symbolized by n, ai, and τi was indicated an excited state fluorescence life-span and the pre-exponential factors. Correspondingly correlated by means of the ith component (Lakowcz, 2006). The chi-square

(χ^2) value indicated the best fitting. The consequential data were evaluated by using the inbuild software of this device.

Quinine sulphate (Φ = 54% in 0.1 N H_2SO_4) was employed as a reference standard to estimation the relative quantum yield of CZ-BT in solution, and it was considered according to literature procedure. The absolute value of quantum yield was calculated by the following formula.

$$Q_{ex} = Q_{st}(A_{ex}/A_{st})(\eta_{ex}/\eta_{st})^2$$

where, Q_{ex} stands for the quantum yield of the experimental samples, A_{ex} and A_{st} were the slop of the integrated emission spectra plotted against absorbance of sample and standard, respectively. η_{ex} and η_{st} indicate the value of the refractive index of the solvent used for dissolving the sample and standard respectively. In time of experiment for both of the samples, excitation wavelength, gain, and slit band width were taken the same value.

2 Materials

2-Bromobenzothiazole and 4-(9H-carbazol-9-yl)phenyl)boronic acid were obtained from bidepharmatech Ltd. tetrakis(triphenylphosphine)palladium, potassium carbonate and N,N-dimethylformamide were purchased from Adamas Reagent Ltd. Acetonitrile was provided by Aladdin Industrial Corporation. Dichloromethane (DCM) and toluene were distilled under normal pressure from calcium hydride under nitrogen immediately prior to use. Tetrahydrofuran (THF) was distilled from sodium/benzophenone under nitrogen. Trichloromethane (TCM) and other commercially available reagents were used as received without further purification.

3 Computational Methods

Geometry optimizations and frequency calculations were performed using density functional theory (DFT) for the ground (S_0) state and time-dependent DFT (TD-DFT) for the lowest excited singlet (S_1) state with the M06-2X functional and def-TZVP basis set. It is known that M06-2X is an ideal choice for main group elements. All above calculations were carried out with the Gaussian 16 B.01 package[1]. Gaussian calculation results are analyzed by Multiwfn[2] 3.8 and VMD[3] 1.9.3 software.

4 Synthesis of CZ-BT

2-Bromobenzothiazole (471 mg, 2.2 mmol), 4-(diphenylamino)phenylboronic acid (574 mg, 2.0 mmol), tetrakis(triphenylphosphine)palladium (27.0 mg, 0.0234 mmol) and 1.0 mL K_2CO_3 saturated solution were added into a 100 mL two necked round-bottom flask. The flask was evacuated under vacuum and flushed with dry nitrogen for three times. Then dry THF (20 mL) was injected. The mixture was heated to 70 °C and stirred for 24 h, and then cooled to room temperature. After evaporating most of THF solvent, the residue was poured into DCM and filtered, the filtrate was collected and then removing the solvent. The crude product was further purified by flash chromatography (silica gel, petroleum ether/DCM, 4/1 to 2/1, v/v), affording a yellow solid with 81.3% yield (612 mg). ^{1}H NMR (500 MHz, DMSO-d_6, δ): 8.46 – 8.25 (m, 4H), 8.25 – 8.09 (m, 2H), 7.96 – 7.82 (m, 2H), 7.73 – 7.42 (m, 6H), 7.34 (t, J = 7.4 Hz, 2H). ^{13}C NMR (126 MHz, DMSO-d_6, δ): ^{13}C NMR (126 MHz, $CDCl_3$) δ 166.92, 154.22, 140.42, 140.15, 135.19, 132.36, 129.08, 127.23, 126.55, 126.19, 125.45, 123.71, 123.39, 121.76, 120.46, 120.43, 109.83. MS (MALDI): calculated for $C_{26}H_{17}NS$ m/z: 376.1034, found m/z: 377.1090 (M+1).

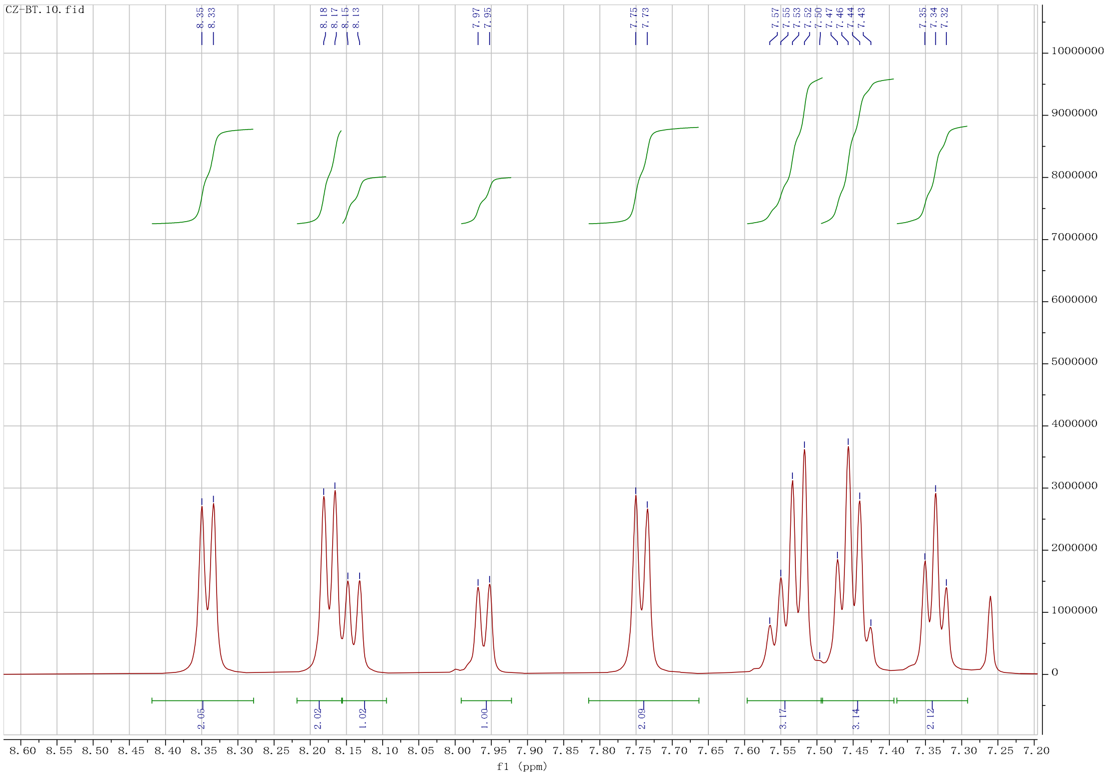

Figure S1 ^{1}H NMR spectrum of CZ-BT.

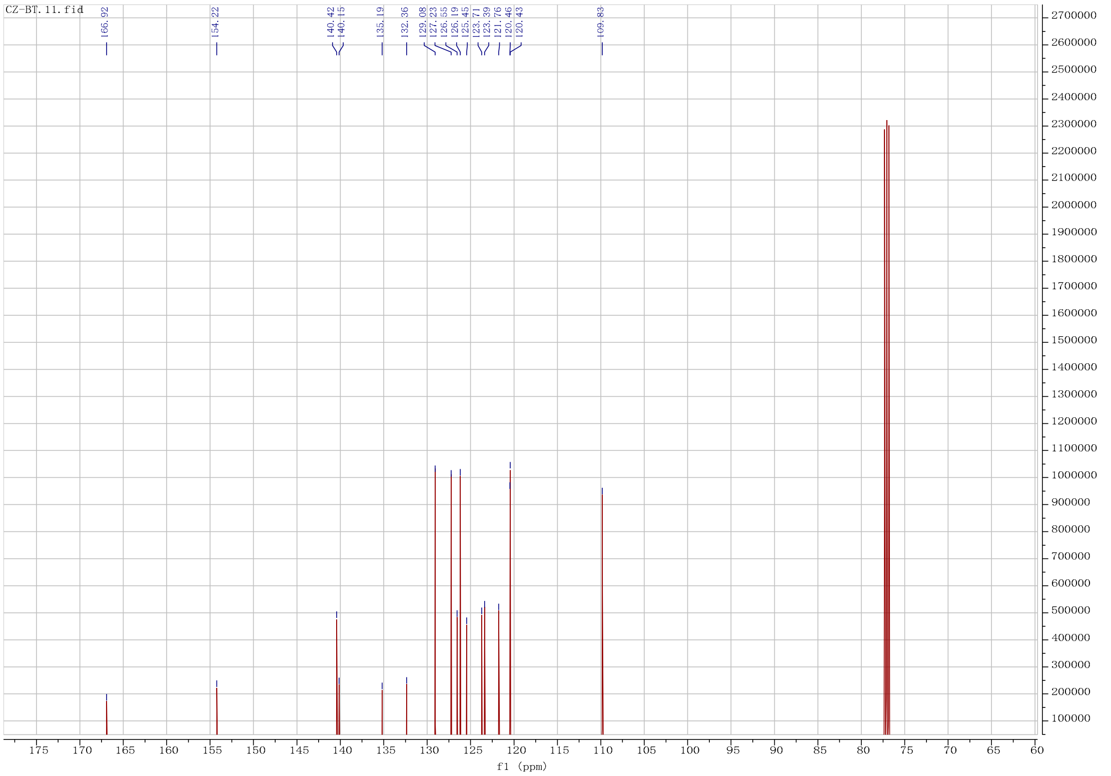

Figure S2 ^{13}C NMR spectrum of CZ-BT.

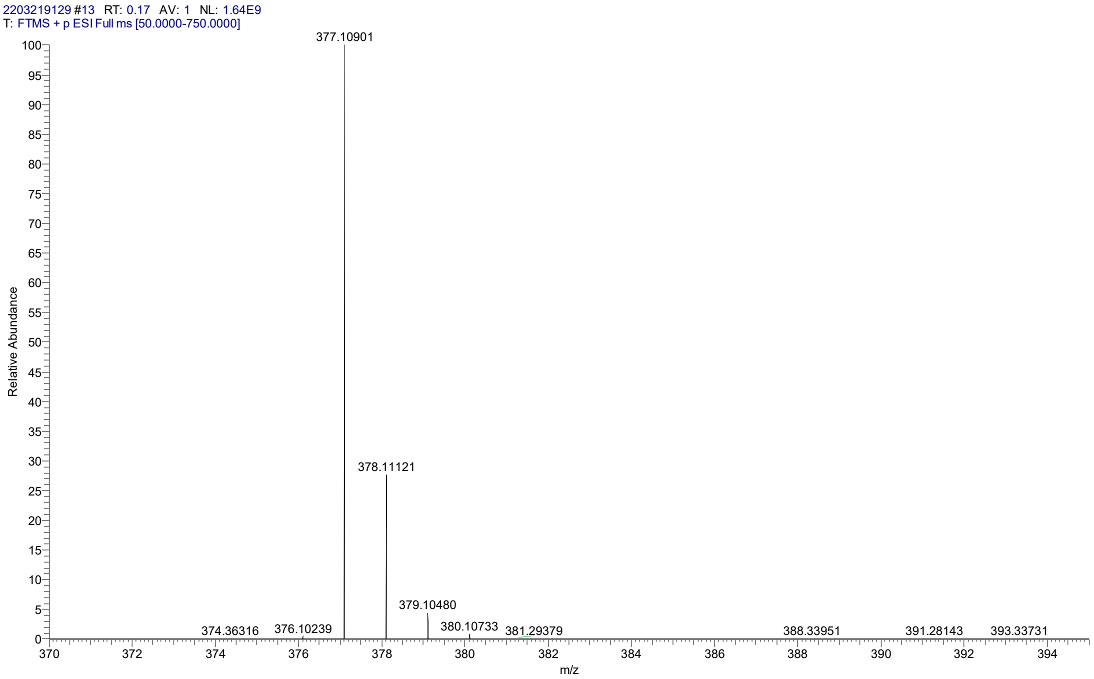

Figure S3 HRMS spectrum of CZ-BT.

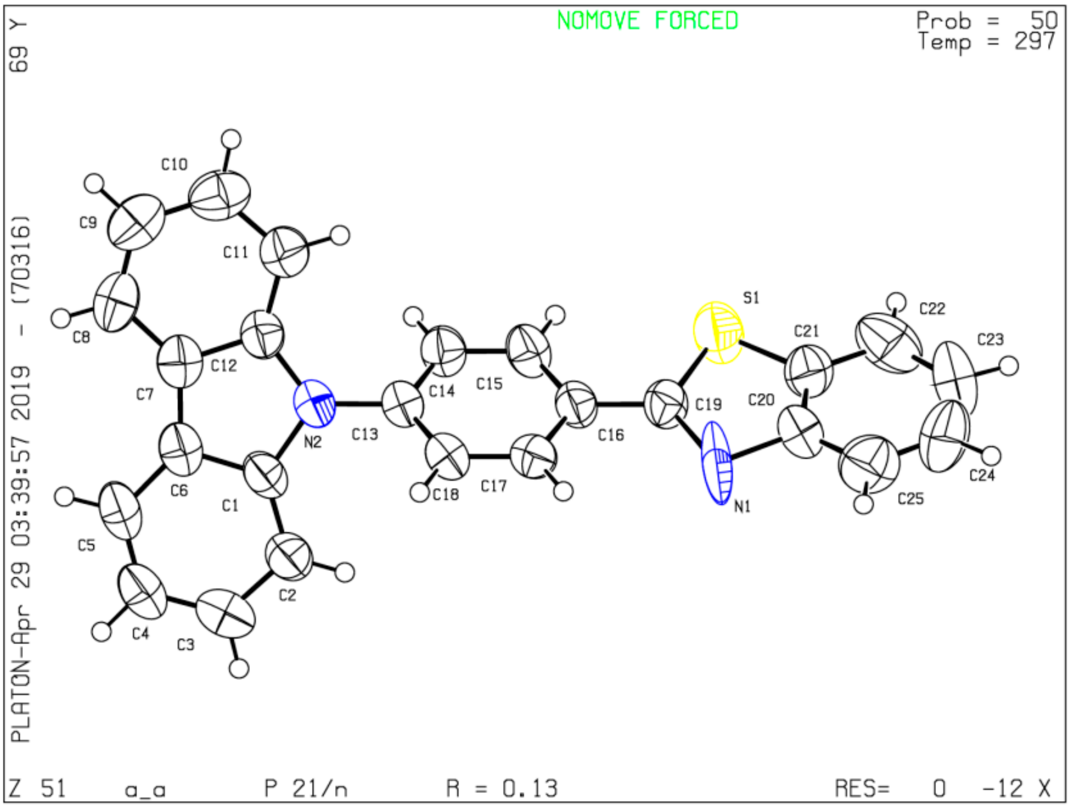

Figure S4 An ORTEP drawing of CZ-BT. Thermal ellipsoids are shown at the 50% probability level.

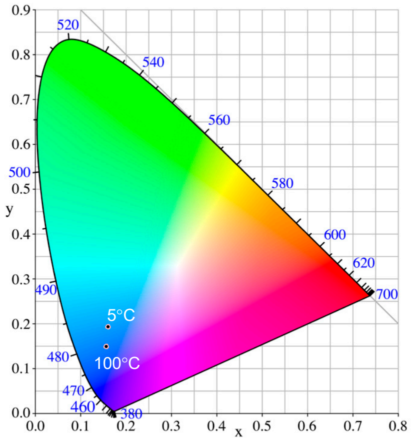

Figure S5 CIE coordinates of DMF solution of CZ-BT at 5 and 100 °C.

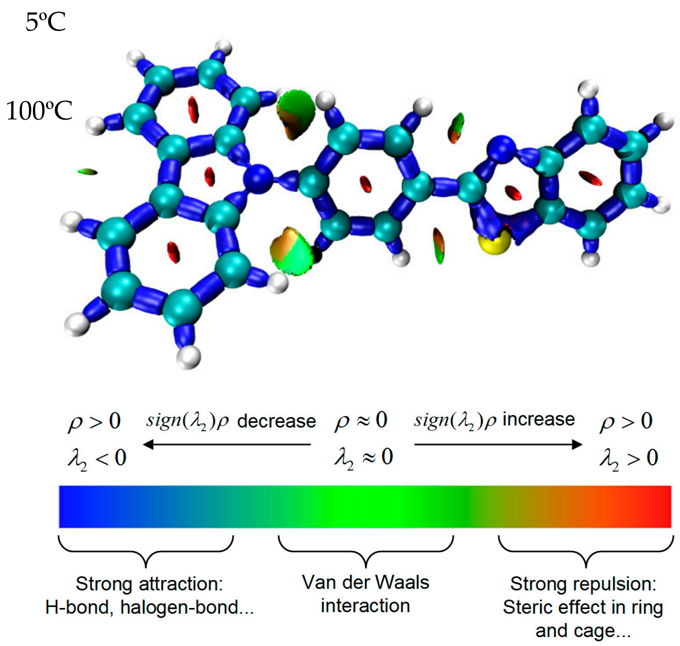

Figure S6 Intramolecular potential hindering effect of CZ-BT.

Table S1 Crystal data and structure refinement for CZ-BT

Identification code	a_a
Empirical formula	C25H16N2S
Formula weight	376.46
Temperature/K	296.87
Crystal system	monoclinic
Space group	P21/n
a/Å	9.7164(17)
b/Å	15.939(3)
c/Å	12.777(2)
α/°	90
β/°	107.253(7)
γ/°	90
Volume/Å3	1889.8(6)
Z	4
ρcalcg/cm3	1.323
μ/mm-1	1.603
F(000)	784
Crystal size/mm3	0.2 × 0.18 × 0.15
Radiation	CuKα (λ = 1.54178)
2Θ range for data collection/°	9.126 to 127.35
Index ranges	-11 ≤ h ≤ 11, -18 ≤ k ≤ 18, -14 ≤ l ≤ 14
Reflections collected	18615
Independent reflections	2973 [Rint = 0.0333, Rsigma = 0.0214]
Data/restraints/parameters	2973/0/253
Goodness-of-fit on F2	2.163
Final R indexes [I>=2σ (I)]	R1 = 0.1282, wR2 = 0.4186
Final R indexes [all data]	R1 = 0.1409, wR2 = 0.4517
Largest diff. peak/hole / e Å-3	1.23/-0.94

Table S2 Colour coordinates of CZ-BT solutions at different temperatures

Temperature/°C	Emission peak/ nm	CIE	
		x	y
5	462	0.1606	0.1936
10	461	0.1603	0.1908
20	459	0.1595	0.1841
30	458	0.1592	0.1803
40	456	0.1588	0.1748
50	455	0.1581	0.1687
60	453	0.1576	0.1643
70	451	0.1571	0.1587
80	450	0.1568	0.1547
90	449	0.1565	0.1513
100	449	0.1563	0.1488

References

1. Frisch, M.J.; Trucks, G.W.; Schlegel, H.B.; Scuseria, G.E.; Robb, M.A.; Cheeseman, J.R.; Scalmani, G.; Barone, V.; Petersson, G.A.; Nakatsuji, H.; et al. *Gaussian 16 Rev. B.01*, Wallingford, CT, 2016.
2. Lu, T.; Chen, F. Multiwfn: a multifunctional wavefunction analyzer. *J. Comput. Chem.* **2012**, *33*, 580-592.
3. Humphrey, W.; Dalke, A.; Schulten, K. VMD: visual molecular dynamics. *J. Mol. Graphics* **1996**, *14*, 33-38.
